# Implementation of corneal confocal microscopy for screening and early detection of diabetic neuropathy in primary care alongside retinopathy screening: Results from a feasibility study

**DOI:** 10.3389/fendo.2022.891575

**Published:** 2022-10-12

**Authors:** Josie Carmichael, Hassan Fadavi, Fukashi Ishibashi, Susan Howard, Andrew J. M. Boulton, Angela C. Shore, Mitra Tavakoli

**Affiliations:** ^1^ Exeter Centre of Excellence for Diabetes Research, National Institute for Health and Care Research (NIHR) Exeter Clinical Research Facility, and Institute of Biomedical and Clinical Sciences, University of Exeter Medical School, Exeter, United Kingdom; ^2^ Peripheral Neuropathy Group, Imperial College, London, United Kingdom; ^3^ University of Manchester, Division of Diabetes, Endocrinology & Gastroenterology, University of Manchester, Manchester, United Kingdom; ^4^ Internal Medicine, Ishibashi Medical and Diabetes Centre, Hiroshima, Japan; ^5^ National Institute for Health Research Collaboration for Leadership in Applied Health Research and Care (NIHR CLAHRC), Manchester, United Kingdom

**Keywords:** screening, diabetic neuropathy, corneal confocal microscopy, early detection, diagnosis

## Abstract

**Objective:**

Screening for diabetic peripheral neuropathy (DPN) is essential for early detection and timely intervention. Quantitative assessment of small nerve fiber damage is key to the early diagnosis and assessment of its progression. Corneal confocal microscopy (CCM) is a non-invasive, in-vivo diagnostic technique that provides an accurate surrogate biomarker for small-fiber neuropathy. In this novel study for the first time, we introduced CCM to primary care as a screening tool for DPN alongside retinopathy screening to assess the level of neuropathy in this novel cohort.

**Research design and methods:**

450 consecutive subjects with type 1 or type 2 diabetes attending for annual eye screening in primary care optometry settings underwent assessment with CCM to establish the prevalence of sub-clinical diabetic peripheral neuropathy. Subjects underwent assessment for neurological and ocular symptoms of diabetes and a history of diabetic foot disease, neuropathy and diabetic retinopathy (DR).

**Results:**

CCM examination was completed successfully in 427 (94.9%) subjects, 22% of whom had neuropathy according to Diabetic Neuropathy Symptom (DNS) score. The prevalence of sub-clinical neuropathy as defined by abnormal corneal nerve fiber length (CNFL) was 12.9%. In the subjects with a short duration of type 2 diabetes, 9.2% had abnormal CNFL. CCM showed significant abnormalities in corneal nerve parameters in this cohort of subjects with reduction of corneal nerve fiber density (CNFD, p<0.001), CNFL (p<0.001) and corneal nerve branch density (CNBD, p<0.001) compared to healthy subjects. In subjects who had no evidence of DR (67% of all subjects), 12.0% had abnormal CNFL.

**Conclusions:**

CCM may be a sensitive biomarker for early detection and screening of DPN in primary care alongside retinopathy screening.

## Introduction

Screening for microvascular complications of diabetes is essential if we are to tackle its devastating complications through early detection and timely intervention. Diabetic retinopathy (DR) screening in the UK has been rated one of the most successful screening programs, in which the NHS offers annual digital fundus photography to all people with diabetes over the age of 12 years (1). This has resulted in diabetes no longer being the leading cause of blindness in the working population among all developed countries (2).

Diabetic peripheral neuropathy (DPN) is the most common and costly diabetes-associated complication, occurring in around 50% of individuals with diabetes (3). It is the strongest initiating risk factor for diabetic foot ulceration. In the UK, people with diabetes account for more than 40% of hospitalizations for major amputations and 73% of emergency admissions for minor amputations (4).

Currently, there is no Food and Drug Administration (FDA) approved therapy to prevent or reverse human DPN (3). The management approach focuses on reasonable glycaemic control, lifestyle modifications, and management of associated pain. Thus, it is important that DPN is detected early in its course. It is recommended that all subjects with type 2 diabetes are screened for DPN at diagnosis, and for type 1 diabetes, the screening should begin five years post-diagnosis (5). After this initial screening, all subjects should be reviewed annually. However, screening for DPN is challenging. There are currently no simple markers for early detection of DPN in routine clinical practice. The measures used are crude and detect the disease late in its natural course.

According to current guidelines for accurate assessment of DPN, a combination of tests may apply which mainly focus on medical history and simple clinical tests such as pinprick and temperature sensation, vibration perception and 10-g monofilament test (6, 7)

While symptoms and neurological deficits have direct relevance to patients, the assessment is excessively variable with poor reproducibility. Similarly, Quantitative Sensory Testing (QST) is subjective, is highly variable, and has limited reproducibility. Neurophysiological tests including Nerve Conduction Studies (NCS) is objective and reproducible but does not assess small fibers, which are the earliest to be damaged and show repair. Small fibers can be assessed objectively by quantifying intra-epidermal nerve fiber density (IENFD) in skin biopsies; however, this is an invasive procedure that requires expert laboratory assessment and results are considerably variable even in healthy controls (8). Therefore, effective treatments may have failed not because of a lack of efficacy, but because of an inability of the currently advocated end points to detect improvement in clinical trials of DN. The use of corneal confocal microscopy (CCM) for rapid, non-invasive clinical assessment of corneal nerves has grown substantially in recent years. It has proven to be particularly useful as a diagnostic marker for the detection of diabetic neuropathy. Numerous studies have confirmed its good sensitivity and specificity (8–14), demonstrating good reproducibility (15) for identifying DPN, proving that it can be particularly useful as a diagnostic marker for screening and stratification of DPN (14, 16–18) as well as a range of other peripheral neuropathies (19–21).

Currently, in the United Kingdom (UK) and globally, there is a lack of robust screening programmes for early detection of DPN and diabetic foot disease, resulting in increasing rates of foot ulcers and amputations among patients with diabetes (22). Previous studies have aimed to establish a screening model for diabetic foot diseases alongside eye disease. These include a recent study in a cohort of patients attending the retinal screening service in a hospital and primary care setting that showed combined eye, foot and renal screening is feasible (23).

The current paper presents the results of a large cohort of subjects screened in primary care using CCM alongside a retinopathy screening service. In comparison to previous CCM studies, this cohort is more representative of the community population for whom CCM could be utilised in the future as a monitoring and screening tool. This group represents subjects, mostly with type 2 diabetes, who are not under hospital care and have less severe complications of diabetes than those who have previously been investigated with CCM. It thus provided an opportunity to explore CCM as a biomarker for DPN in a novel cohort.

## Materials and methods

### Study group

The current dataset was originally collected as part of a study for investigating the feasibility and acceptability of implementing CCM for screening of DPN in primary care alongside the diabetic retinopathy screening program in South Manchester Diabetic Retinopathy Screening Service (SMDRSS) at four primary care optometry practices (24). During the six-month study (2014–2015), 450 consecutive subjects with type 1 or type 2 diabetes attended annual diabetes eye screening. Of those who consented and enrolled on the study, 95.5% had type 2 diabetes, 38% were female, and the median age of the whole study population was 68 years (range 21-93). The median duration since diabetes diagnosis was 6 years (0.1-51).

**Figure 1:**
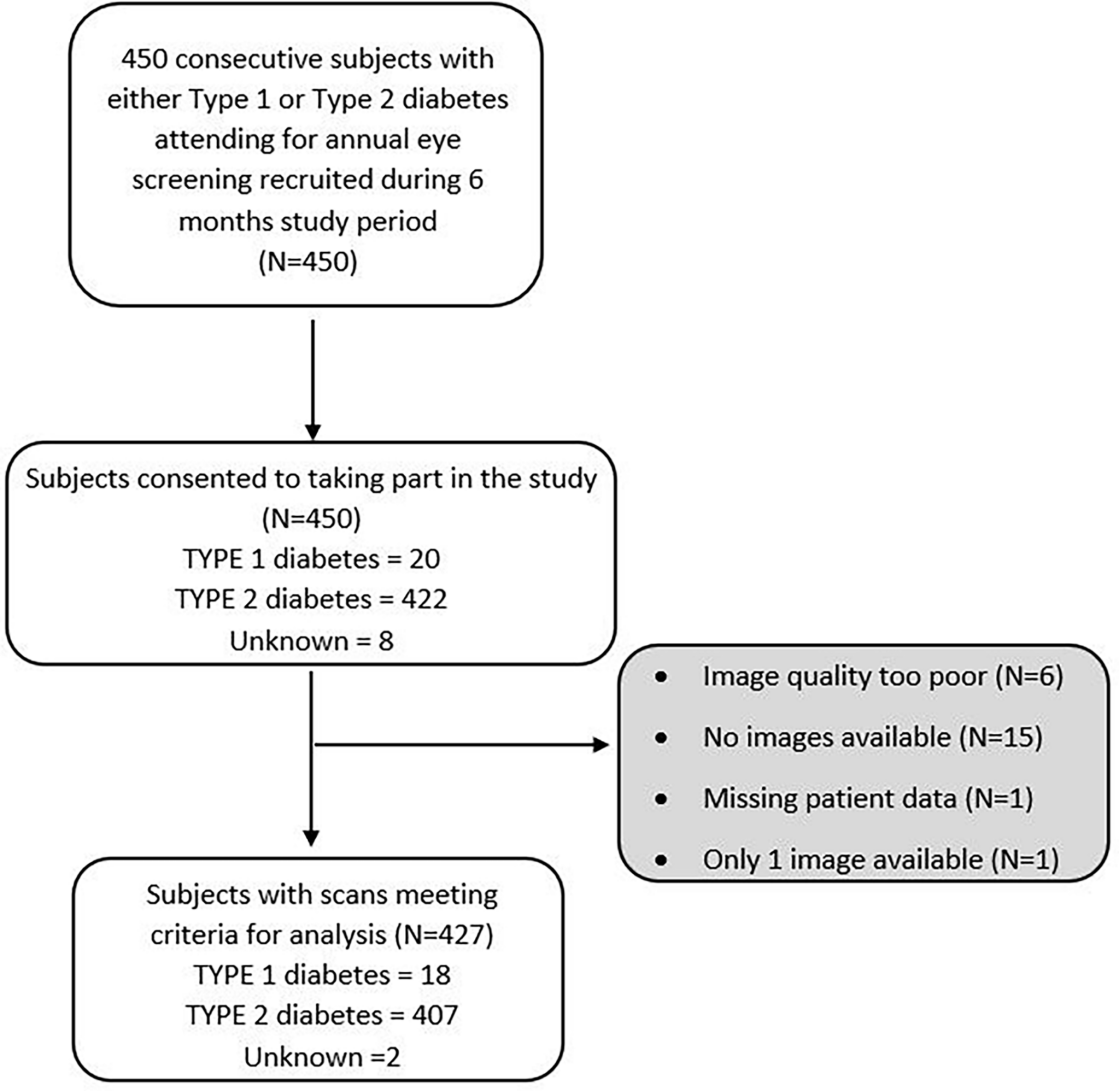
Study flow diagram.

Data were successfully collected to an acceptable standard from 427 subjects (**Figure 1** and **Table 1**) by four experienced and trained optometrists. The composition of the study population was compared against the UK population with diabetes as reported in the National Diabetes Audit (NDA) (25). Overall, apart from ethnicity, the composition of the study population was similar to the UK population with diabetes for age, gender, type of diabetes and duration of diabetes.

**Table 1:** Characteristics of the cohort with diabetes.

		Subgroups			
	All Subjects with Diabetes (N = 427)	Type 1 Diabetes (N = 18)	Type 2 Diabetes(N = 407)	Type 2 Diabetes ≤2 yrs. (N = 98)	No Retinopathy (R0) or Maculopathy (M0) (N = 241)
**Gender** Female n (%) Male n (%)	167 (39%) 260 (61%)	8 (44%) 10 (56%)	158 (39%) 249 (61%)	39 (40%) 59 (60%)	94 (39%) 147 (61%)
**Age, years**	67.9 (21-93)	67.9 (21-93)	68.30 (21-93)	60.85 (21-89)	68.40 (34-92)
**Type of Diabetes** Type 1 n (%) Type 2 n (%) Unknown	18 (4%) 407 (95%) 2 (<1%)	–	–	–	3 (1%) 236 (98%) 2 (1%)
**Duration of diabetes, years**	6 (0.1-51)	6 (0.1-51)	6 (0.1-30)	1 (0.1-2)	5 (0.10-51)
**Ethnicity** White n (%) Black n (%) Asian n (%) Mixed Other/unknown	347 (81%) 65 (15%) 12 (3%) 2 (1%) 3 (1%)	16 (89%) 2 (11%) 0 0 0	328 (81%) 63 (15%) 12 (3%) 2 (1%) 2 (1%)	79 (81%) 15 (15%) 3 (3%) 1 (1%) 0	202 (83.5%) 31 (13%) 5 (2%) 1 (0.5%) 2 (1%)
**History of DN** Yes n (%) No n (%) Unknown	28 (6.5%) 397 (93%) 2 (0.5%)	3 (17%) 15 (83%) 0	25 (6%) 380 (93%) 2 (1%)	4 (4%) 94 (96%) 0	8 (3%) 231 (96%) 2 (1%)
**History of Foot Ulcer** Yes n (%) No n (%) Unknown	16 (4%) 410 (96%) 1 (<0.5%)	3 (17%) 15 (83%) 0	13 (3%) 393 (97%) 1 (<0.5%)	2 (2%) 96 98%) 0	4 (1.5%) 236 (98%) 1 (0.5%)
**History of Retinopathy** Yes n (%) No n (%) Unknown	146 (34%) 273 (64%) 8 (2%)	5 (28%) 13 (72%) 0	133 (33%) 266 (65%) 8 (2%)	20 (20%) 77 (79%) 1 (1%)	0 241 (100%) 0
**History of Laser Treatment for Retinopathy** Yes n (%) No n (%) Unknown n (%)	9 (2%) 417 (98%) 1 (<0.5%)	2 (11%) 16 (89%) 0	7 (2%) 399 (98%) 1 (<0.5%)	1 (1%) 97 (99%) 0	0 241 (100%) 0
**DNS Score** 0 n (%) 1 n (%) 2 n (%) 3 n (%) 4 n (%)	262 (61%) 73 (17%) 49 (12%) 21 (5%) 22 (5%)	14 (78%) 3 (16.5%) 1 (5.5%) 0 0	246 (60.5%) 70 (17%) 48 (12%) 21 (5%) 22 (5.5%)	71 (73%) 13 (13%) 7 (7%) 3 (3%) 4 (4%)	163 (68%) 34 (14%) 22 (9%) 11 (4.5%) 11 (4.5%)
**Retinopathy Grading** R0 n (%) R1 n (%) R2 n (%) R3 n (%)	288 (67%) 132 (31%) 4 (1%) 3 (1%)	5 (28%) 10 (55.5%) 1 (5.5%) 2 (11%)	281 (69%) 122 (30%) 3 (1%) 1 (<0.5%)	78 (80%) 20 (20%) 0 0	–
**Maculopathy Grading** M0 n (%) M1 n (%)	414 (97%) 13 (3%)	17 (94%) 1 (6%)	395 (97%) 12 (3%)	95 (97%) 3 (3%)	–
**CNFD** (no./mm²) Semi-automated Automated	25.84 (± 7.08) 21.63 (± 7.10)	23.79 (± 9.75) 18.31(± 9.72)	25.94 (± 6.95) 21.76 (± 6.95)	27.65 (± 6.96) 23.59 (± 6.91)	26.28 ( ± 7.02) 21.98 ( ± 7.00)
**CNBD** (no./mm²) Semi-automated Automated	75.00 (0-212.50) 29.16 (0-82.29)	64.58 (5-119.79) 22.39 (2.08-49.96)	75.00 (0-212.50) 29.16 (0-82.29)	79.16 (0-194.79) 31.25 (4.17- 82.29)	78.12 (0-210.42) 30.21 (0-82.29)
**CNFL** (mm/mm²) Semi-Automated Automated	19.37 (± 5.68) 13.62 (± 3.55)	17.29 (± 6.73) 12.80 (± 4.32)	19.48 (± 5.63) 13.68 (± 3.52)	20.78 (± 5.45) 14.45 (± 3.66)	19.78 ( ± 5.53) 13.80 ( ± 3.46)
**TC** (TC)	16.90(9.60-32.66)	15.39(9.46-32.66)	17.00(8.18-31.67)	16.42(10.86-31.33)	16.73 (8.85 - 29.72)
**CNFW** (mm/mm²)	0.021 (0.019-0.030)	0.022 (0.020-0.028)	0.021 (0.019-0.030)	0.022 (0.021-0.023)	0.021 (0.019-0.030)
**CNFA** (mm²/mm²)	0.006 (0.001-0.013)	0.005 (0.003-0.008)	0.006 (0.001-0.013)	0.005 (0.002-0.010)	0.006 (0.002-0.011)
**CTBD** (no./mm²)	46.87 (0-138.5)	34.50 (12.50-78.12)	46.87 (0-138.5)	62.50 (59.37-65.62)	47.91 (6.25-123.95)
**LCs Presence** Yes n (%) No n (%)	417 (98%) 10 (2%)	417 (98%) 10 (2%)	397 (98%) 10 (2%)	97 (99%) 1 (1%)	234 (97%) 7 (3%)
**LCs Density** (no./mm²)	22.92 (0-225)	40.31 (2.08-126.04)	22.92 (0-225)	28.65 (0-225)	20.83 (0-170.38)

Summary of the characteristics of the whole cohort with diabetes (Subjects), the subjects with type 1 diabetes, subjects with type 2 diabetes, subjects with type 2 diabetes and less than, or equal to, two years since disease diagnosis, and subjects with no evidence of retinopathy (R0) or maculopathy (M0). Age and duration of diabetes are represented by median (range) due to non-normal distribution. Retinopathy grading based on the ETDRS criteria: 0 = no retinopathy, 1 = background, 2 = pre-proliferative, 3 = proliferative. Maculopathy grading: 0 = no maculopathy 1 = maculopathy. ‘Unknown’ represents subjects for whom information was not available. Excluded cases were those with image quality that was deemed unacceptable, or there were < 2 images available for analysis.

The research adhered to the tenets of the Declaration of Helsinki, and ethical approval was granted by the NRES East Midlands committee (REC: 15/EM/0079). All subjects provided written informed consent before participating in this study. Inclusion criteria were subjects aged 16 years and over with type 1 or 2 diabetes participating in NHS diabetes retinopathy screening programme. The exclusion criteria were subjects under 16, subjects who were unable to provide written consent, concurrent ocular disease, ocular infection or inflammation which may affect the cornea, a history of ocular disease or systemic disease that has affected the cornea (e.g. keratoconus, corneal dystrophies, refractive surgery), and wearing a hard contact lens. Full details of the study methods can be found in the National Institute for Health Research (NIHR) report (24).

### History of neuropathy, foot ulcer and retinopathy

All attending subjects completed a detailed questionnaire on their history of diabetic complications. Specifically, they were asked about any previously diagnosed neuropathy, diabetic foot disease, foot ulceration, gradable retinopathy, and previous laser treatment for DR.

### Assessment of neuropathy

The Diabetic Neuropathy Symptom Score (DNS) was used to assess each subject with diabetes for clinically evident DPN. The DNS score is a four-item symptom score for assessing DPN, developed by an expert panel and has been described previously (26). A score of 1 or more, out of a maximum of 4, indicates clinically detectable DPN (26).

### Retinopathy screening

All subjects underwent retinal screening in one of the four primary care optometry practices. A detailed description of the Diabetic Retinopathy Screening Service (DRSS) (1) and the retinopathy grading criteria (27) have been published previously. In summary, retinopathy is graded as one of four grades from R0 to R3. R0 represents no DR, R1 represents background DR, R2 represents pre-proliferative DR, and R3 represents proliferative DR. Mydriatic, 2-dimensional fundus photography was carried out on each patient. Images were graded by the appropriately qualified, attending optometrist for the level of DR with reference to previously established criteria (27).

### Examination with CCM

Each subject underwent assessment using in-vivo CCM (Heidelberg Retinal Tomograph III Rostock Cornea Module; Heidelberg Engineering GmbH, Heidelberg, Germany) by a trained optometrist, based on established methodology (28). Each optometrist selected eight (4 per eye) high-quality, non-overlapping images from Bowman’s layer and sub-basal nerve plexus in a masked, randomized order. Through a secure server that was established and supported by the University of Manchester, the fully anonymized images were shared on a daily basis with the PI (MT). An in-depth protocol of this imaging technique has been described previously (24).

### CCM analysis

Images of the corneal sub-basal nerve plexus were evaluated for quality and artefacts by the attending optometrist. Six non-overlapping masked images were analysed per subject using semi-automated CCMetrics software (MA Dabbah; Imaging Science and biomedical engineering, University of Manchester, Manchester, UK). It is recognised that semi-automated analysis can be a time consuming, resource intensive procedure as it involves manual tracing of nerve fibres (29). Considering this, fully automated, algorithmic defined, software such as ACCMetrics (University of Manchester, Manchester, UK) has been developed to eliminate the manual input. However, some small cohort studies have previously reported problems of automated software such as false positive and false negative identification of nerve structures (30, 31) and systematically lower measures of CNFL compared to semi-automated methods, despite good correlation (29). We therefore included both methods of analysis.

The following semi-automated CCM parameters were quantified: (i) Corneal nerve fiber density (CNFD) – the total number of major nerves/mm^2^ of corneal tissue, (ii) corneal nerve branch density (CNBD) – the number of branches emanating from all major nerve trunks/mm^2^ of corneal tissue, (iii) corneal nerve fiber length (CNFL) – the total length of all nerve fibers and branches (mm/mm^2^), (iv) tortuosity coefficient (TC) and (v) Langerhans cells (LCs) presence and density within the area of corneal tissue.

The ACCMetrics automated software produces three parameters which are comparable to the semi-automated software (CNFL, CNFD and CNBD), as well as three additional parameters: corneal total branch density (CTB) - the total number of branch points from the main nerve/mm^2^,corneal nerve fiber area (CNFA) - total nerve fiber area (mm^2^/mm^2^), and corneal nerve fiber width (CNFW) - the average nerve fiber width (mm/mm^2^).

### Statistics

Statistical analysis was performed in Microsoft Excel for Office 365 (Microsoft Corp, Seattle, WA, USA) and SPSS for Windows version 26 (SPSS Inc., Chicago, IL, USA). Data were tested for normal distribution, and appropriate statistical tests were carried out accordingly.

For agreement analysis of automated vs semi-automated software, a two-way, mixed-effects model intraclass correlation test was conducted for absolute agreement between patient values. This was expressed as intraclass correlation coefficient (ICC) and 95% confidence interval.

A sample size of 400 subjects with a 95% confidence interval of ±5% was originally calculated to allow estimations of the proportions on study outcomes. This decision was made in consideration of equipment availability and the feasibility for the practices to recruit enough participants during the timeframe of the original study.

## Results

### Demographics and clinical information

The demographics, relevant patient history and clinical information for the subjects are presented in **Table 1**. The patient population of this study purposefully included wide ethnic and socio-economic mix factors, according to the NIHR deprivation index. This was broadly comparable to the adult national population with diabetes.

From the 450 subjects recruited for the study, 23 (5.1%) were excluded. **Figure 1** displays reasons for exclusion.

### Correlation with age

Overall, there was a significant negative age-related correlation for semi-automated CNFD, CNFL and CNBD (p<0.001 for all 3) (**Table 2**). Similarly, there was a significant negative correlation between age and all three parameters measured using automated software (p<0.001 for CNFL and CNFD, p=0.002 for CNBD). The only other parameter with a significant negative correlation to age was corneal total branch density (CTBD) derived using automated software. There was no correlation of tortuosity (using either coefficient), LCs density, corneal nerve fiber width (CNFW) and corneal nerve fiber area (CNFA) with age.

**Table 2:** Correlations of nerve fiber parameters with age.

Statistic			Age (Years)	
		n	Rs	p-value
**CNFD**: Corneal nerve fiber density	Semi-automated	427	-0.26	<0.001
(no/mm^2^)	Automated	427	-0.27	<0.001
**CNBD**: Corneal nerve branch density	Semi-automated	427	-0.2	<0.001
(no./mm^2^)	Automated	427	-0.15	0.002
**CNFL**: Corneal nerve fiber length	Semi-automated	427	-0.24	<0.001
(mm/mm^2^)	Automated	427	-0.2	<0.001
**TC**: Tortuosity coefficient	(0-1)	427	0.07	0.10
	(0-20)	427	0.07	0.20
**LCs: Langerhans cells Density** (no./mm^2^)		427	0.09	0.06
**CTBD**: Corneal total branch density (no./mm^2^)		427	-0.12	0.02
**CNFA**: Corneal nerve fiber area (mm^2^/mm^2^)		427	0.002	>0.90
**CNFW**: Corneal nerve fiber width (mm/mm^2^)		426	0.02	0.70

Spearman’s correlation (Rs) and statistical significance (p-value) of semi-automated and automated CCM image analysis with age. Data analyzed from patient cohort and included subjects with type 1 and type 2 diabetes. Significant correlations are highlighted in red.

### Comparison of corneal nerve data derived using semi-automated and automated analysis of the same CCM images

**Figure 2:**
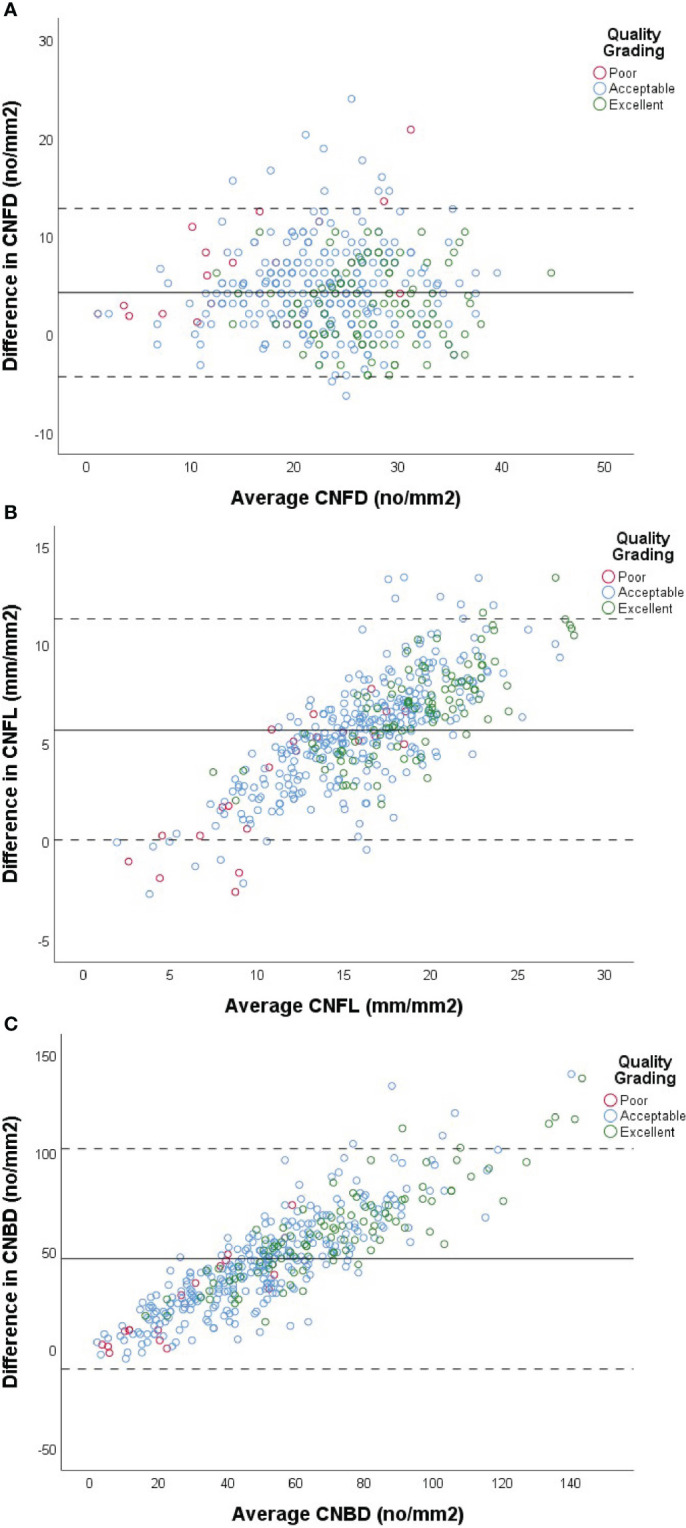
Comparison of automated vs semi-automated analysis of CNFD **(A)**, CNFL **(B)** and CNBD **(C)** using Bland-Altman plots. X-axis of each plot represents the mean measurement between the two methods of analysis for each subject. Y-axis represents the difference in values between the two methods for each subject, calculated by (semi-automated value - automated value) for each patient. Data from cohort with diabetes used in analysis (n=427). Solid line represents the mean difference between the two methods. Dashed lines represent +/- (1.96 x 2SD). Red, blue and green markers represent images graded as poor, acceptable and excellent by the investigator, respectively.

In order to compare agreement between automated and semi-automated corneal nerve analysis, values for CNFD, CNFL and CNBD for each method were assessed (**Table 3**). For most of the subjects examined (88.3%), a higher measurement for CNFD was obtained using semi-automated analysis in comparison to the automated method (**Figure 2A**). Overall, the mean difference between the two measurements was 4.26 (no/mm^2^), and the mean percentage difference was 16.49%, with a mean higher value for semi-automated analysis. ICC values gave moderate agreement (ICC=0.75) between the two measures. There was no correlation between the mean CNFD number and the difference between the two measurements (**Figure 2A**).

**Table 3:** Comparison of automated and semi-automated analysis.

		CNFD (no./mm^2^)	CNFL (mm/mm^2^)	CNBD (no./mm^2^)
**Semi-automated Analysis**	Mean	25.83	19.35	77.3
	±SD	7.08	5.69	37.92
	SEM	0.34	0.28	1.83
			
**Automated Analysis**	Mean	21.57	13.62	30.96
	±SD	7.11	3.56	16.45
	SEM	0.34	0.17	0.79
			
	Mean Difference	4.26	5.73	46.34
	Mean % Difference	16.49	29.61	59.95
	P-value	<0.001	<0.001	<0.001
	ICC	0.75	0.63	0.41
	95% Confidence Interval	0.039-0.912	-0.183-0.876	-0.204 - 0.723

Comparison of automated and semi-automated quantified measurements for CNFD, CNFL, and CNBD. Analysis conducted on the cohort with diabetes (n = 427). Results reportedas mean, standard deviation (SD) and standard error of the mean (SEM). Mean difference value represents the mean difference between results from each method. Mean % difference represents the average difference expressed as a percentage of the semi-automated analysis result (i.e. if the semi-automated CNFL result for a parameter was 20mm/mm2 and for automated software is was 10mm/mm2 then the % difference would be 50% as the difference is 50% of the semi-automated CNFL). Two-way mixed models for ICC are shown with 95% confidence intervals and statistical significance reported to represent agreement between semi-automated and automated software. All p-values calculated with a paired samples T-test. Statistical significance determined by p ≤0.05.

For most subjects (97.0%), a higher measurement for CNFL was obtained using semi-automated analysis in comparison to automated software (**Figure 2B**). Overall, the mean difference between the two measurements was 5.73(mm/mm^2^), Overall, the mean difference between the two methods for CNBD was 46.34 (no/mm2) (**Figure 2C**) and the mean percentage difference between the two measurements was 29.61%, with an overall higher mean for semi-automated analysis. ICC values again gave moderate agreement (ICC =0.63) between the two measures; however, confidence intervals for ICC values were broad (**Table 3**). There was a modest positive correlation between mean CNFL and the difference between the two measurements (**Figure 2B**), indicating that the discrepancy between the methods becomes greater as the fiber length increases.

Overall, the mean difference between the two methods for CNBD was 46.34 (no/mm^2^), and the mean percentage difference was 59.95%, making CNBD the parameter with the largest % disagreement between the two methods. ICC values gave poor agreement (ICC=0.41) between the two methods, and confidence intervals for ICC values were broad (**Table 3**). There was modest positive correlation between mean CNBD and the difference between the two measurements (**Table 3**).

### Prevalence of small fiber neuropathy

As the results demonstrated age-related negative correlations, we assessed whether subjects’ CCM data differed from those expected in healthy individuals, using data from a published age-segregated normative range by an international consortium (32). Due to the large differences between our automated and semi-automated results, we used only our semi-automated for comparison to the semi-automated derived published normative ranges. **Table 4** displays the median values for the male and female subjects with diabetes, separated into six groups, based on age at the point of examination. Data are presented for semi-automated analysis derived CNFD and CNFL, compared to each age group’s published normative median values. All age groups in the cohort of male individuals with diabetes demonstrate a median value for CNFL less than the normative published data. This was also true for all but one of the age groups in the female cohort, where the median of the 56-65 age group was 0.57 mm/mm^2^ higher than that of the published normative median. Due to very small numbers of subjects under the age of 45, we assessed the number of subjects whose CNFL was less than the normally published median for their age group. For the female cohort, 7 of the 8 subjects under the age of 45 had a CNFL length that was less than the median. For the male cohort, 10 of the 13 subjects under the age of 45 had a CNFL length that was less than the median.

**Table 4:** Comparison to normative age-related median and cut-off values.

			CNFD			CNFL					
	Age	n	Cohort median	Normative median	Difference	Cohort median	Normative median	Difference	CNFL Cut-off value (mm/mm2)	< CNFL Cut-off (no)	< CNFL Cut-off (%)
**FEMALES**	16-25	2	22.40	31.85	9.45	16.07	26.43	10.36	15.08	1	50.00
**(n = 167)**	26-35	1*	20.83	30.20	9.37	13.24	25.45	12.21	13.17	0	0.00
	36-45	5	26.04	28.56	2.52	21.84	24.37	2.53	12.48	1	20.00
	46-55	29	30.21	26.91	**-3.3**	21.87	23.28	1.41	12.48	1	3.45
	56-65	36	27.08	25.27	**-1.81**	22.77	22.20	**-0.57**	12.9	3	8.33
	>65	94	23.96	23.54	**-0.42**	18.86	21.11	2.25	13.67	14	14.89
**Total**		**167**								**Type 1: 2 Type 2: 18 Overall: 20**	**Type 1: 25 Type 2: 11.39 Overall: 11.98**
**MALES**	16-25	1*	23.96	32.44	8.48	17.73	23.16	5.43	15.93	0	0
**(n =260)**	26-35	3	25.00	30.56	5.56	18.81	22.92	4.11	14.05	1	33.33
	36-45	9	26.04	28.68	2.64	17.57	23.34	5.77	13.20	1	11.11
	46-55	26	29.69	26.80	**-2.89**	22.94	23.63	0.69	13.01	3	11.54
	56-65	66	28.04	24.92	**-3.12**	20.23	23.03	2.80	13.12	7	10.61
	>65	155	25.00	22.95	**-2.05**	18.66	20.61	1.95	13.15	23	14.84
**Total**		**260**								**Type 1: 2 Type 2: 33 Overall: 35**	**Type 1: 20 Type 2: 13.25 Overall: 13.46**

Comparison of 2 semi-automated corneal nerve parameters (CNFD and CNFL) with age-matched published normative values (32) for females and males. ‘Cohort Median’ represents the median value in each age group of the patient cohort. ‘Normative Median’ represents the published median values for females in each age group (32). The difference between the normative and cohort medians was calculated as (Normative median – Cohort median). Positive values are represented in red, whereas negative values are shown in black. (* unable to calculate median as n = 1). Classification of females and males within as having pathological CNFL when compared to published cut-off values (25) (0.05th quantile of normative database). The number and % of subjects classified as having pathological CNFL is given for each age group.

For both males and females, the median CNFD was lower than the normative published median CNFD for the three youngest age groups; 16-25, 26-35 and 36-45. Again, due to small numbers of subjects under the age of 45, we assessed the number of subjects whose CNFD was less than the normally published median for their age group. For the female cohort, 6 of the 8 subjects under the age of 45 had a CNFD that was less than the median. For the male cohort, 9 of the 13 subjects under the age of 45 had a CNFD that was less than the median. In contrast, the median CNFD was higher than the normative published median CNFD for males and females in the three oldest age groups; 46-55, 56-65 and over 65.

Age-corrected values at which CNFL may be considered abnormal have previously been published (32). When compared to these values, 20 (11.98%) females were below the CNFL cut-off (**Table 4**) and classified as abnormal. Of these 20 females, 2 had type 1, and 18 had type 2 diabetes. Overall, 25% of subjects with type 1 diabetes and 11.39% with type 2 diabetes were classified as abnormal.

A slightly higher proportion of 35 (13.46%) males in the patient cohort were below the CNFL cut-off. Of these 35 males, 2 had type 1 diabetes, and 33 had type 2 diabetes. Overall, 20% of males with type 1 diabetes and 13.25% of males with type 2 diabetes were classified as abnormal using CNFL alone.

### CNFL in subjects based on diabetes disease duration

In order to assess whether there may be corneal nerve alterations early in the course of diabetes for this cohort, a sub-group of the subjects who were diagnosed with diabetes ≤ 2 years ago were compared with age-corrected normal published values for CNFL (**Tables 1**, **5**). Due to the very small number of subjects with type 1 diabetes (n=2), these subjects were excluded, and those with type 2 diabetes were considered alone for these analyses (n=98). When assessing the group of subjects with ≤ 2 years duration of diabetes from the time of diagnosis, 9.18% of subjects were classified as below the age-corrected published cut-off point for CNFL (32) and would have been considered abnormal for this parameter alone.

**Table 5:** Comparison of groups based on years since diagnosis.

	≤ 2 Years	2-5 Years	5-10 Years	10-20 Years	>20 Years	p-value
n	98	83	131	82	13	-
**Age (years)**	60.85 (21-89)	63.30 (34-87)	69.10 (45-92)	72.45 (46-93)	77.20 (57-86)	<0.001
**Gender** F M	39 (40%) 59 (60%)	32 (39%) 51 (61%)	53 (40%) 78 (60%)	27 (33%) 55 (67%)	7 (54%) 6 (46%)	
**Ethnicity** White Black Asian Mixed Other	79 (81%) 15 (15%) 3 (3%) 1 (1%) 0	65 (78%) 15 (18%) 3 (4%) 0 0	110 (84%) 16 (12%) 2 (1.5%) 1 (1%) 2 (1.5%)	65 (79%) 13 (16%) 4 (5%) 0 0	9 (69%) 4 (31%) 0 0 0	–
**DNS Score** 0 1 2 3 4	71 (72.5%) 13 (13.5%) 7 (7%) 3 (3%) 4 (4%)	51 (61.5%) 12 (14.5%) 6 (7%) 10 (12%) 4 (5%)	70 (53.5%) 29 (22.5%) 20 (15%) 3 (2%) 9 (7%)	45 (55%) 15 (18%) 13 (16%) 4 (5%) 5 (6%)	9 (69%) 1 (8%) 2 (15%) 1 (8%) 0	–
**Retinopathy Grade** R0 R1 R2 R3	78 (80%) 20 (20%) 0 0	63 (76%) 20 (24%) 0 0	94 (72%) 36 (27%) 1 (1%) 0	41 (50%) 39 (48%) 2 (2%) 0	5 (38%) 7 (54%) 0 1 (8%)	–
**Maculopathy Grade** M0 M1	95 (97%) 3 (3%)	82 (99%) 1 (1%)	130 (99%) 1 (1%)	77 (94%) 5 (6%)	11 (85%) 2 (15%)	–
**No of subjects < CNFL cut-off**	9 (9.18%)	10 (12.05%)	18 (13.74%)	11 (12.20%)	2 (15.38%)	

Summary of the known characteristics and clinical grading information for subjects with type 2 diabetes, split into 5 age groups and control subjects (Controls). Age is represented by median (range) due to a non-normal distribution. Retinopathy grading: 0 = no retinopathy, 1= background, 2 = pre-proliferative, 3 = proliferative. Maculopathy grading: 0 = no maculopathy 1 = maculopathy. See methods section for detailed grading characteristics. ‘Unknown’ represents subjects for which information was not available. Number of subjects <cut-off was calculated using published age-corrected values (32).

When considering CNFL in participants with a longer duration of diabetes (**Table 5**), the percentage of patients falling below the normative published cut-off value were similar across the middle three duration groups (12.05-13.74%). The group with the shortest duration (≤ 2 years) had a smaller percentage of patients falling below the cut-off value (9.18%) and the group with the longest duration of diabetes (>20 years) had the highest percentage of patients falling below the cut-off value (15.38%) however due to the small number of patients in each group, we were unable to test for statistical significance.

### CCM parameters in subjects with different grades of retinopathy

**Figure 3:**
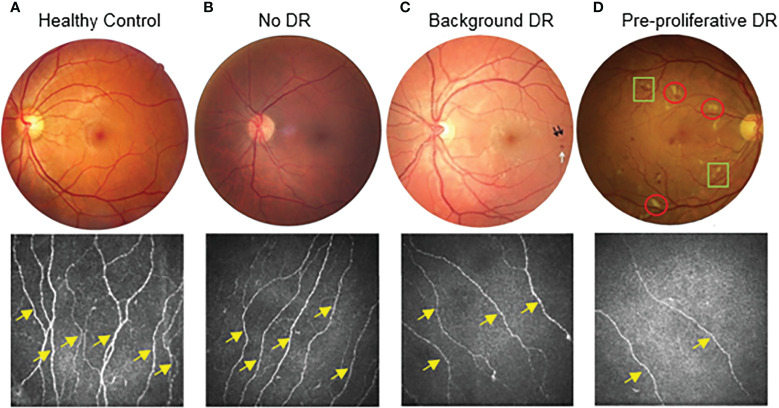
Stages of diabetic retinopathy linked to CCM findings from healthy control to pre-proliferative diabetic retinopathy (DR grade 2). Proliferative (DR grade 3) not displayed. **(A)** demonstrates a healthy control subject. **(B)** demonstrates a patient with diabetes but no diabetic retinopathy (DR grade 0). **(C)** demonstrates a patient with background diabetic retinopathy (DR grade 1). Dot haemorrhages (black arrows) and a blot haemorrhage(white arrow) can be seen. **(D)** demonstrates a patient with diabetes and pre-proliferative diabetic retinopathy (DR grade 2). As well as multiple dot and blot haemorrhages, there are also numerous cotton wool spots (red circles) and intra-retinal microvascular abnormalities (IRMA)(green squares). For the corresponding examples of CCM images, the nerve fibre density of the main nerves (yellow arrows) decreases as diabetic retinopathy progresses. There is also a clear decrease in the overall nerve fibre length.

CNFD was compared across four groups according to DR grading (**Figure 3** and **Table 6**). When testing for significance between groups R0 and R1, a non-significant difference was found (p=0.37). Three of the four subjects (75%) in the R2 group had a CNFD level less than the published normative median value for their age group. For group R3 subjects, all three subjects (100%) had a CNFD level which was less than the published median normative value for their age group. As the number of subjects in groups R2 and R3 was very small, we could not perform statistical tests.

**Table 6:** Comparison of different retinopathy grades.

Retinopathy grade	R0	R1	R2	R3	p-value
n	288	132	4	3	-
**Age (years)**	68.35 (23-92)	67.50 (21-93)	65.90 (30-69)	50.40 (41-77)	0.50
**Type of diabetes** Type 1 Type 2 Unknown	5 (2%) 281 (97.5%) 2 (0.5%)	10 (7.5%) 122 (92.5%) 0	1 (25%) 3 (75%) 0	2 (66.5%) 1 (33.5%) 0	
**Duration of diabetes**	6 (0.10-51)	9 (0.20-35)	14 (6-20)	21 (11-35)	<0.001
**Gender** F M	109 (38%) 179 (62%)	54 (41%) 78 (59%)	3 (75%) 1 (25%)	1 (33.5%) 2 (66.5%)	0.50
**Ethnicity** White Black Asian Mixed Other	233 (81%) 42 (14.5%) 9 (3%) 2 (0.5%) 2 (0.5%)	106 (80.5%) 23 (17.5%) 3 (2%) 0 0	4 (100%) 0 0 0 0	3 (100%) 0 0 0 0	0.70
**DNS Score** **0** **1** **2** **3** **4**	186 (64.5%) 44 (15%) 30 (10.5%) 14 (5%) 14 (5%)	72 (55%) 29 (22%) 18 (13.5%) 7 (5%) 6 (4.5%)	2 (50%) 0 0 0 2 (50%)	2 (66.5%) 0 1 (33.5%) 0 0	0.20
**Maculopathy Grade** M0 M1	286 (99.5%) 2 (0.5%)	125 (94.5%) 7 (5.5%)	0 4 (100%)	3 (100%) 0	–
**CNFD (no/mm^2^)**	26.18 (± 7.03)	25.53 (± 6.90)	23.70 (± 6.66)	11.04 (± 6.38)	–
**CNBD (no/mm^2^)**	77.08 (0-212.50)	71.87 (0-161.46)	43.23 (25.0-122.92)	28.12 (5.0-50.0)	–
**CNFL (mm/mm^2^)**	19.70 (± 5.65)	18.93 (± 5.57)	18.06 (± 5.44)	8.36 (± 4.23)	–
**No of subjects < CNFL cut-off**	34 (11.81%)	18 (13.64%)	0	3 (100%)	

Summary of the known characteristics and clinical grading information for subjects, assorted into 4 groups, based on retinopathy grade, as well as controls. The CCM parameters are calculated with Semi-automated software. Age and duration of diabetes are represented by median (range) due to a non-normal distribution. Retinopathy grading: 0 = no retinopathy, 1 = background, 2 = pre-proliferative, 3 = proliferative. Maculopathy grading: 0 = no maculopathy 1 = maculopathy. See methods section for detailed grading characteristics. ‘Unknown’ represents subjects for which information was not available. The number of subjects below cut-off was calculated using published age-corrected values (32).

Similarly, CNFL demonstrated no significant difference between the R0 and R1 subjects (p=0.19). As published, suggested cut-off points are available for CNFL; these were used to assess each group. In the R0 group (**Tables 1**, **6**), 11.81% of subjects with R0 fell below the cut-off point. This increased to 13.64% in the R1 group and 100% in the R3 group; however, none of the R2 group fell below the CNFL cut-off point.

According to the Early Treatment Diabetic Retinopathy Study (ETDRS), grading of DR (31), R0 and M0 represent no detectable retinopathy and maculopathy, respectively. Therefore, the group of subjects meeting these criteria and with no previous history of retinopathy, maculopathy or laser was compared with age-corrected normal published values for CNFL to assess any significant changes prior to detectable retinopathy (**Table 1**). The patient group consisted of mainly subjects with type 2 diabetes (98%). The majority of the patient group had a DNS score of 0 (68%), with 32% scoring positively on the DNS score for at least one symptom of neuropathy. Based on CNFL length alone, 12.0% of subjects were below the age-dependent published cut-off point, suggesting that 12.0% of subjects with no evidence of retinopathy may have significant CNFL reduction.

## Discussion

This study assessed the implementation of CCM to screen for DPN in clinical practice outside of the research environment and to our knowledge, is the first study of this type. As a measure of the corneal sub-basal nerve plexus, CCM provides a potential surrogate biomarker for assessing small nerve fiber changes in subjects with diabetes. Several studies that recruited subjects from hospital clinics have confirmed CCM’s ability to detect nerve alterations in people with diabetes compared to healthy controls (5, 6, 8, 11, 15) and distinguish between subjects with and without clinical DPN (9, 12). CCM has shown promise for predicting future neuropathy from baseline measurements (11) and has detected nerve regeneration post-therapeutic intervention (33).

Automated software is significantly quicker when analysing images in comparison to semi-automated software. It is likely the only viable option for analysis if using CCM to screen for neuropathy in the future. In our study, when comparing automated and semi-automated analysis, the results for automated CNFD, CNFL and CNBD were all significantly lower. This is in agreement with previous studies, also finding an underestimation when using ACCMetrics automated software (29, 31) and was the reason we focused mainly on semi-automated methods for the most accurate analysis. If automated software is to be used for DPN screening, software needs to be improved and updated to resolve the measurements bias. As we await these technological advances, adjustment factors must be put in place to compare to semi-automated analysis.

In this study, we found that CNFL, CNFD and CNBD significantly decreased with increasing age (**Table 2**), in line with previously published literature (32, 34). Thus, we referenced CCM published normative age values for CNFL and CNFD (32). Twenty females (11.98%) and 35 (13.46%) males were classified as having abnormal CNFL that could be considered clinically significant (12.88% overall). This implies that in our cohort, 12.9% of subjects may be deemed to have small fiber neuropathy if using CNFL as a single diagnostic measure. This percentage is less than that of Anderson et al. (2018) (35), who found a prevalence of 19% DPN when using the Toronto consensus for diagnosis in subjects with type 2 diabetes. CCM identifies small fiber damage, which has been shown to precede large fiber changes (36, 37); thus, we expected a higher percentage of abnormality in our study. This highlights the problematic nature of comparing DPN prevalence across studies using a range of definitions for classification. The Diabetes Control and Complications Trial (DCCT) data exemplified the impact of varying diagnostic testing procedures. In their cohort, the prevalence of DPN at baseline varied from 0.3% (abnormalities of reflexes, sensory examination and neuropathic symptoms) to 21.8% (abnormal nerve conduction in at least two nerves) depending on the criteria used for detection (38).

A recent study of 236 people attending retinal screening showed that combined eye, foot and renal screening is feasible. In that study, the authors reported a prevalence of DPN, assessed using the Toronto Clinical Neuropathy Score, of 30.9%, which was underestimated by the 10-g monofilament test (14.4%). The clinical characteristics of the cohorts might explain the differences between their findings and our results, as studies are never identical with respect to the demographics of their subjects and risk factors for DPN. The authors do not report the duration of diabetes of their patient group (23), and additionally, subjects attended screening either in primary care or within a hospital setting. Subjects that attended the secondary care setting may have been at higher risk of diabetic complications. An important aspect of our study was that subjects were tested during community screening. Although this would need to be confirmed with further studies, it is likely that the relative stability of subjects attending community retinopathy screening would make them less susceptible to developing diabetic complications such as DPN and associated reduction in corneal nerve fibers.

To assess the potential role of CCM to identify early nerve changes, it was important to evaluate subjects with diabetes of duration ≤ 2 years since diagnosis. This was to determine if corneal changes were occurring early in diabetes.

When assessing subjects with diabetes within early stages since diagnosis, Ziegler and colleagues (18) concluded, using their own control cohort, that CNFD was the most sensitive parameter for detecting neuropathy, as it detected 21% of subjects falling below the 2.5th percentile of the control group. CNFL was the second most sensitive, with 17% falling below the 2.5th percentile (18). This percentage for CNFL abnormality is significantly higher than that of abnormal CNFL in subjects with diabetes, found in our study (9.18%). It is likely that the significantly lower comparative percentage is largely due to a difference in percentile cut-off points used to define an abnormality. In comparison, we used the 0.5^th^ percentile as a cut-off point from age-corrected published values (32), therefore identifying fewer subjects as outside of this range.

It is difficult to confidently compare the results of these two studies as although sample sizes were similar (86 vs 98), our study evaluated only subjects with ≤ 2 years disease duration, whereas the mean disease duration of the subjects in the Ziegler et al. (2014) study was 2.1± 1.6 years. The longer duration of diabetes in some of their cohort may have caused more significant corneal nerve changes.

One recently published study (39) found that there was no significant difference in CNFL between patient groups with type 2 diabetes duration <10 years (mean age 5 ± 3) and control subjects. This contradicts the findings of our study and that of Ziegler and colleagues (18); however, it may be attributed to the study’s strict inclusion/exclusion criteria - subjects with glycated haemoglobin (HbA1c) levels of >7.8% or a history of proliferative retinopathy were excluded.

Despite limited research into subjects during the very early stages of diabetes, our findings suggest that corneal nerve fiber changes may occur early and may be an indicator of changes in the sensory nervous system.

At present, retinal photography is a successful screening method for DR and can detect early microvascular changes. Our findings suggest that changes in corneal nerves may precede detectable retinopathy.

These findings confirm those of Bitirgen et al. (40), who reported, in subjects with type 2 diabetes and no DR, a significant reduction in CNFD (p<0.001), CNFL (p=0.02) and CNBD (p =0.001) compared to healthy subjects when assessed using automated software. An earlier study (41) also found a significant difference in all three parameters; however, this study used their own custom-written routines in MATLAB rather than a commonly used software such as CCMetrics.

When assessing subjects with type 1 diabetes, two similar studies (42, 43) reported a reduction in CNFD, CNFL and CNBD, prior to any retinopathy. However, Szalai et al. (2016) only assessed young subjects (mean age 22.86 ± 9.05 years), which was not representative of the type 1 diabetes population overall. This cohort was very different to ours, which was (1) mainly in people with type 2 diabetes and (2) of significantly older age.

Our study into this area is novel in that we assessed subjects in primary care along with DR screening. This has allowed us to evaluate a larger cohort of 241 subjects with no retinopathy or history of retinopathy compared to previous studies. Of these subjects, 29 (12.0%) had a CNFL measurement less than the published age-corrected reference value. This may suggest that several subjects do not meet the referral criteria into the hospital eye service (HES) based on retinopathy but may require further investigation and closer monitoring of peripheral nerve changes. More studies are needed to investigate the cost-effectiveness of this increase in referrals and the benefits to the subjects.

Although our study demonstrates good agreement with the current literature, the four previous studies discussed were completed in a hospital setting by a trained expert, thus were not representative of a cohort attending community DR screening. There was also a significant lack of recently diagnosed subjects (< 2 years), most notably in one of these studies (41). Nevertheless, the findings of these and our studies challenge the current screening strategies deployed to detect the complications of diabetes. Using CCM to identify corneal nerve changes may be the earliest window of opportunity to intervene and prevent the progression of the triad of microvascular complications; nephropathy, neuropathy and retinopathy.

There were some limitations to this study: first, there was no available information regarding height, triglyceride levels or HbA1c levels, which were previously associated with increased risk of neuropathy (44). A recent study by Wang et al. (2020) (45) found that subjects with type 2 diabetes and DPN had significantly higher levels of HbA1c (p=0.035), high-density lipoprotein (p=0.003) and fasting blood glucose (p=0.026). We are unable to confidently conclude that any significant/non-significant changes between subgroups of subjects were down to the grouping factor and no other independent factors.

Second, our cohort was made up of mainly older subjects with type 2 diabetes. This may be considered partially as a limitation, as we were unable to perform statistical testing on data from younger subjects and subjects with type 1 diabetes. However, the composition of the study population was compared against the UK population with diabetes; thus, our cohort mirrors the demographic of subjects attending the retinal screening service in the UK and therefore adequately acts as a representative population of this specific group.

Due to frequent delays in diabetes diagnosis in primary care, the exact time of disease onset is uncertain. One study previously reported a delay of at least 4-7 years before diagnosing type 2 diabetes (46). Subjects in our study classed as having the disease duration of ≤ 2 years may be wildly different from the precise time since disease onset, thus erroneously suggesting more significant changes to corneal nerve fibers early in the course of diabetic disease.

Finally, due to the nature of this study, we were unable to assess the neuropathy in detail, including nerve conduction studies (NCS) to use as an objective assessment of DPN and comparator to determine the sensitivity and specificity of CCM to diagnose DPN. However, the sensitivity and specificity of CCM for said measurement has been previously validated in a number of studies (8–12, 14, 47–49).

To our knowledge, this study has been the first to use CCM to assess a large cohort of subjects with diabetes in a primary care screening service in which CCM images were taken by primary care clinicians. Our study presents evidence that CCM can be used in primary care to accurately detect corneal nerve abnormalities prior to evident retinopathy and in the early years since diagnosis. Overall, the findings support the current literature that CCM is a sensitive surrogate biomarker for DPN. Further research should focus on developing software for automated analysis and validating its diagnostic validity for detecting early DPN in larger, age-matched cohorts in primary care.

## Data availability statement

The raw data supporting the conclusions of this article will be made available by the authors, without undue reservation.

## Ethics statement

The studies involving human participants were reviewed and approved by NRES East Midlands committee (REC: 15/EM/0079). The patients/participants provided their written informed consent to participate in this study.

## Author contributions

JC and MT wrote the manuscript. JC, HF, FI, MT contributed to data analysis and image analysis of the study subjects. JC, HF, FI, AB, SH, AS, MT reviewed and revised the paper. MT and AS supervised JC. MT was the PI study. MT designed, conceived the study, wrote the article, major revisions, made comments, had full access to all data, and is the guarantor. All authors provided important intellectual input and approved the final version of the manuscript. All authors contributed to the article and approved the submitted version.

## Funding

This research was funded by the National Institute for Health Research Collaboration for Leadership in Applied Health Research and Care Greater Manchester, now recommissioned as NIHR Applied Research Collaboration Greater Manchester.

## Acknowledgments

The authors thank the Manchester NIHR/Wellcome Trust Clinical Research Facility and Exeter NIHR Clinical Research Facility for their support. The views in the manuscript are those of the authors and not those of the NIHR or the Department of Health and Social Care. Special thanks to the National Institute for Health Research Collaboration for Leadership in Applied Health Research and Care (NIHR CLAHRC) Greater Manchester Team, especially to Prof. Ruth Boaden, Ms. Rebecca Spencer, Ms. Claudia Soiland-Reyes, Dr Sarah Cotterill, Ms. Catherine Perry. We would particularly like to thank the optometrists and reception staff who generously gave their time to deliver this study. Special thanks to participated optometrists: Mr. Stephen Epstein (Scotts Optician), Mr. Joe Arrowsmith (Arrowsmith Eyecare), Mr. Suhail Hakim (Alan Miller Optometrists), and Mr. Suhayl Issa (Murrays Opticians). We would like to thank Heidelberg Engineering for loaning their specialist CCM equipment and help to deliver the clinical training. Special thanks to the Heidelberg Engineering team, Krysten Williams, Chris Mody, Melanie Polzer, and Phil Enion, for their continuous support. Special thanks to Mr. Dipesh Mehta from the University of Manchester for the IT support. And finally, our gratitude goes to all subjects and participants in this study.

## Conflict of interest

The authors declare that the research was conducted in the absence of any commercial or financial relationships that could be construed as a potential conflict of interest.

## Publisher’s note

All claims expressed in this article are solely those of the authors and do not necessarily represent those of their affiliated organizations, or those of the publisher, the editors and the reviewers. Any product that may be evaluated in this article, or claim that may be made by its manufacturer, is not guaranteed or endorsed by the publisher.

## Abbreviations

CCM, Corneal Confocal Microscopy; CNBD, Corneal Nerve Branch Density; CNFA, Corneal Nerve Fiber Area; CNFD, Corneal Nerve Fiber Density; CNFL, Corneal Nerve Fiber Length; CNFW, Corneal Nerve Fiber Width; CTBD, Corneal Total Branch Density; DNS, Diabetic Neuropathy Score; DPN, Diabetic Peripheral Neuropathy; HbA1c, Glycated Hemoglobin; LC; Langerhans Cells; NIHR, National Institute for Health Research; TC, Tortuosity Coefficient.
